# Bladder cancer detection in urine using DNA methylation markers: a technical and prospective preclinical validation

**DOI:** 10.1186/s13148-022-01240-8

**Published:** 2022-02-05

**Authors:** Anouk E. Hentschel, Irene J. Beijert, Judith Bosschieter, Paul C. Kauer, André N. Vis, Birgit I. Lissenberg-Witte, R. Jeroen A. van Moorselaar, Renske D. M. Steenbergen, Jakko A. Nieuwenhuijzen

**Affiliations:** 1 Department of Urology, Amsterdam UMC, Vrije Universiteit Amsterdam, Amsterdam, The Netherlands; 2 Department of Pathology, Cancer Center Amsterdam, Amsterdam UMC, Vrije Universiteit Amsterdam, Amsterdam, The Netherlands; 3 Department of Urology, OLVG, Amsterdam, The Netherlands; 4 Epidemiology and Data Science, Amsterdam UMC, Vrije Universiteit Amsterdam, Amsterdam, the Netherlands

**Keywords:** Biomarkers, Tumour, DNA methylation, Liquid biopsy, Urinary bladder neoplasms, Urine

## Abstract

**Background:**

The development of accurate urinary biomarkers for non-invasive and cost-effective detection of primary and recurrent bladder tumours is recognized as one of the major clinical needs in bladder cancer diagnostics**.** The purposes of this study were (1) to validate the results of a previous technical comparison by determining the diagnostic performance of nine methylation markers in urine pellet compared to full void urine, and (2) to validate the diagnostic performance of the optimal marker panel *GHSR/MAL* from a previous exploratory study in a preclinical setting.

**Methods:**

Urine samples of 108 patients with bladder cancer and 100 age- and gender-matched controls were prospectively collected for methylation analysis. Urinary methylation levels of the markers *FAM19A4*, *GHSR*, *MAL*, *miR-129*, *miR-935*, *PHACTR3*, *PRDM14*, *SST* and *ZIC1* were determined with quantitative methylation-specific PCR in urine pellet. Area under the curves (AUCs) were determined for individual markers and the marker panel *GHSR/MAL*. The diagnostic performance of the marker panel *GHSR/MAL* was evaluated in the total study population and in different subgroups of patients with bladder cancer using the Chi-square test. The diagnostic accuracy was assessed by leave-one-out cross-validation.

**Results:**

All nine urinary methylation markers (*FAM19A4, GHSR, MAL, miR-129, miR-935, PHACTR3, PRDM14, SST* and *ZIC1*) showed significantly higher methylation levels in bladder cancer patients than in controls (*p* < 0.001). Area under the curves (AUCs) of the nine methylation markers tested in urine pellet were similar to AUCs in full void urine of an independent previous cohort. *GHSR/MAL* reached an AUC of 0.89 (95% confidence interval [CI] 0.84–0.94), at 80% sensitivity and 93% specificity. Sensitivity of *GHSR/MAL* increased with higher tumour grades, higher tumour stages, in primary vs. recurrent tumours, and in males vs. females.

**Conclusions:**

This technical validation supports the robustness of DNA methylation analysis in urine pellet and full void urine for the non-invasive detection of bladder cancer. Subsequent preclinical validation confirmed the diagnostic potential of *GHSR/MAL*. These findings underline the diagnostic potential of the marker panel *GHSR/MAL* for future bladder cancer diagnostics.

**Supplementary Information:**

The online version contains supplementary material available at 10.1186/s13148-022-01240-8.

## Introduction

The presence of a bladder tumour is often discovered after episodes of painless macroscopic haematuria. At initial diagnosis, the disease is non-muscle-invasive in approximately 75% of patients [1]. Since non-muscle-invasive bladder cancer (NMIBC) has the tendency to recur or progress to muscle-invasive disease, regular and long-term cystoscopic evaluations are mandatory [1]. Yet, cystoscopy has important disadvantages as it is an invasive procedure that is associated with high costs [2]. Urine cytology is used as a complementary and non-invasive tool in patients with high-grade (HG) urothelial bladder tumours, but sensitivity is limited in low-grade (LG) disease [1, 3]. Besides its suboptimal sensitivity, the use of urine cytology is hampered by its subjective interpretation [4]. Therefore, research focuses on the identification of reliable urinary biomarkers to allow for objective, non-invasive and cost-effective detection of bladder cancer [5, 6].

Multiple studies have reported on the potential of DNA methylation markers as urinary biomarkers for bladder cancer diagnostics [7]. DNA hypermethylation in promoter regions of tumour suppressor genes can lead to inactivation of their tumour suppressive function, accordingly contributing to the development of cancer. As DNA hypermethylation is considered to be among the earliest events in urothelial carcinogenesis, DNA methylation analysis poses a promising tool for the early detection of bladder tumours [8]. In a previous exploratory study, DNA methylation analysis in full void urine of patients with bladder cancer (*n* = 72) and healthy controls (*n* = 75) identified ten genes with significantly higher urinary methylation levels in patients with bladder cancer compared to healthy controls [9]. The diagnostic performance proved best for the marker panel *GHSR/MAL*, reflected by an area under the curve (AUC) of 0.89, at 92% sensitivity and 85% specificity. In a subsequent technical comparison of urine fractions with the nine most discriminative methylation markers, it was demonstrated that urine pellet is preferred over full void urine or urine supernatant [10]. Urine pellet represented the respective tumour tissues best, it reached the highest discriminative capability for the marker panel *GHSR/MAL*, and it was the most convenient to process.

The aims of the present study are (1) to validate the results of our technical comparison by determining the diagnostic performance of these nine methylation markers (*FAM19A4, GHSR, MAL, miR-129, miR-935, PHACTR3, PRDM14, SST* and *ZIC1*) in urine pellet compared to full void urine and (2) to validate the diagnostic performance of the best performing marker panel from our exploratory study (*GHSR/MAL*) in a preclinical setting.

## Materials and methods

### Patients

**Fig. 1:**
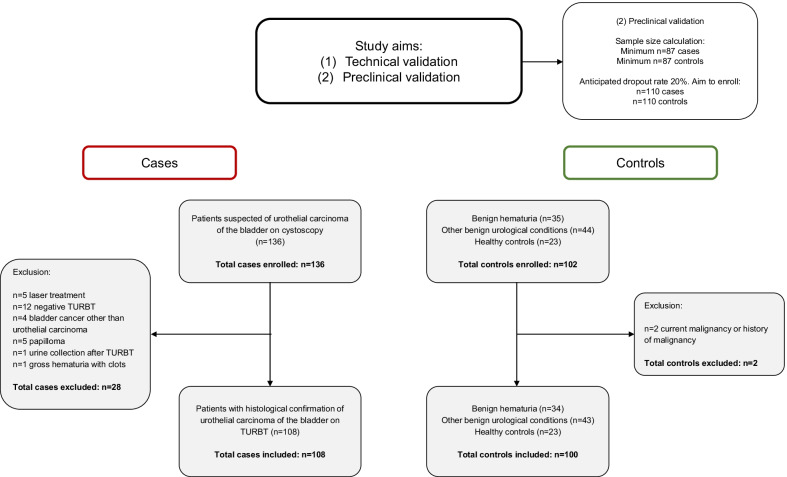
Outline of the study and flowchart for patient inclusion

Patients were prospectively included between October 2018 and October 2020 at Amsterdam University Medical Centers and at OLVG. Patients suspected of urothelial carcinoma of the bladder on cystoscopy were eligible for inclusion. Presence of urothelial carcinoma of the bladder had to be confirmed by transurethral resection of the bladder tumour (TURBT). Patients were excluded if no TURBT was performed or if TURBT did not show evidence of urothelial carcinoma of the bladder. Controls were excluded if they had a current malignancy or a history of malignancy. The outline of the study and flowchart for patient inclusion are shown in Fig. 1. DNA methylation analysis was performed in 108 patients with bladder cancer and 100 controls who were matched based on age and gender (Table 1). Of the patients with bladder cancer, 70% had a primary tumour and the disease was non-muscle invasive in 79%. Histological tissues were graded following the World Health Organization (WHO) 1973 (grades 1–3 [G1–G3]) and 2004/2016 (LG-HG) classification systems. Controls consisted of patients who were evaluated for haematuria (*n* = 34), patients who were diagnosed with other benign urological conditions (*n* = 43) and healthy controls (*n* = 23). The study was conducted in accordance with the Declaration of Helsinki, and the study protocol was approved by the Medical Ethics Committee (urology: 2018.355 [16-10-2018], WO 18.155 [21-12-2018]; healthy controls: 2018.657 [07-02-2019]). All participants gave informed written consent for study participation prior to inclusion.

**Table 1:** Baseline characteristics of the study population

Characteristics	Bladder cancer (*n* = 108)	Controls (*n* = 100)	*p *value
Age, *yr, (IQR)*	71 (63–75)	66 (59–75)	0.25
*Gender, n (%)*			0.12
Male	79 (73)	63 (63)	
Female	29 (27)	37 (37)	
*WHO 1793, n (%)*			
G1	17 (16)	–	
G2	39 (36)	–	
G3	52 (48)	–	
*WHO 2004/2016, n (%)*			
LG	45 (42)	–	
HG	63 (58)	–	
*Tumour stage, n (%)*			
Ta	59 (55)	–	
T1	16 (15)	–	
Tis	10 (9)	–	
≥ T2	23 (21)	–	
*Primary/recurrence, n (%)*			
Primary	76 (70)	–	
Recurrence	32 (30)	–	
*Number of tumours, n (%)*			
Solitary	59 (55)	–	
Multiple	49 (45)	–	
*Tumour size, n (%)*			
< 3 cm	55 (51)	–	
≥ 3 cm	43 (40)	–	
Unknown	10 (9)	–	
*Concomitant CIS, n (%)*			
No	97 (90)	–	
Yes	11 (10)	–	
*Controls, n (%)*			
Benign haematuria	–	34 (34)	
Other benign urological conditions	–	43 (43)	
Healthy controls	–	23 (23)	

*CIS* carcinoma in situ, *G* grade, *HG* high-grade, *IQR* Interquartile range, *LG* low-grade, *WHO* World Health organization, *yr* year

### Urine samples

Patient samples were collected before cystoscopy or TURBT and were processed within 24–72 h after collection. DNA quality was preserved by the addition of 0.6 M ethylenediaminetetraacetic acid in a final concentration of 40 mM [11]. Urine samples were pelleted by centrifugation of 15 mL urine at 800×*g* for 10 min, and thereafter stored at − 20 °C.

### DNA isolation, bisulphite conversion and quantitative methylation-specific PCR

DNA isolation was performed with QIAamp DNA Mini Kit (Qiagen GmbH, Hilden, Germany). DNA concentrations were measured with NanoDrop 1000 (ThermoFisher Scientific, Waltham, MA, US). Next, bisulphite conversion was achieved with EZ DNA Methylation™ Kit (Zymo Research, Orange, CA, USA). DNA isolation and bisulphite conversion were in accordance with the manufacturer’s protocols. Quantitative methylation-specific PCR (qMSP) was conducted for the target genes in three different multiplex assays (*FAM19A4/PHACTR3/PRDM14/ACTB; GHSR/SST/ZIC1/ACTB*; *MAL/miR-129/miR-935/ACTB*) as described previously [9, 12]. qMSPs were performed with 50 ng of bisulphite-converted DNA as input. The methylation values of the target genes were normalized for the reference gene *ACTB* by using the comparative Ct method (2^−∆CT^ × 100) to obtain Ct ratios. In six patients (2.9%; one bladder cancer patient, five controls), results of all target genes from one or more multiplexes were considered invalid due to an *ACTB* Ct > 32 [13].

### Statistical analyses

For the sample size calculation, the area under the curve (AUC) of the marker panel *GHSR/MAL* from the previous exploratory study was used (AUC 0.89) [9]. To obtain a maximum width of 0.1 for the two-sided 95% confidence interval (CI), a minimum sample size of *n* = 87 patients with bladder cancer and *n* = 87 controls was required. As a dropout rate of 20% was anticipated for cases and controls, we aimed to enrol *n* = 110 participants in both groups (Fig. 1).

Categorical data were described with frequencies and percentages, and continuous data with medians and interquartile range (IQR). The Chi-square test was performed to compare categorical data between patients with bladder cancer and controls. The Mann–Whitney *U* test was used to compare means of continuous data between both groups. The methylation levels of the urinary markers were calculated as log2-transformed Ct ratios and were visualized as boxplots. The Kruskal–Wallis test was used to compare the methylation levels of the urinary markers between controls with benign haematuria, other benign urological conditions and healthy controls. Since the methylation levels did not differ between the control cohorts, all 100 controls were considered as one single group. The Ct ratios of the urinary methylation markers were used to plot a receiver operating characteristic (ROC) curve and to establish the AUC. The level of discrimination of an AUC of 0.5–0.6 was considered very poor, 0.6–0.7 poor, 0.7–0.8 fair, 0.8–0.9 good, and 0.9–1.0 excellent [14].

The diagnostic performance of the marker panel *GHSR/MAL* was scored positive if at least one of both markers was positive (‘believe-the-positive’) [9, 15]. Due to the use of urine pellet instead of full void urine [9], new thresholds were determined for the marker panel *GHSR/MAL* with Youden’s J index (Additional file 1: Table S1) [16, 17]. Leave-one-out cross-validation (LOOCV) was used to evaluate the accuracy of the diagnostic performance of the marker panel *GHSR/MAL*. In addition, a random forest analysis was performed to further establish the ability of all nine markers to distinguish between cases and controls.

The sensitivity of the marker panel *GHSR/MAL* was also determined with respect to tumour grade (G3 vs. G1–G2, and HG vs. LG), tumour stage (≥ T2 vs. Ta/T1/Tis), disease status (primary vs. recurrent) and gender (male vs. female). The Chi-square test was used to assess the differences in diagnostic performance between these subgroups of patients with bladder cancer. Based on the previous and current differences in the sensitivity of the marker panel *GHSR/MAL* for gender, post hoc analyses were performed with males and females considered as separate cohorts [9]. New thresholds of the Ct ratios were determined for both genders with Youden’s J index (Additional file 1: Table S1) [16, 17].

Sample size calculation was performed with PASS version 15.0. Statistical analyses were done with SPSS Software (SPSS 26.0, IBM Corp., NY, USA) and R Statistical Software (version 3.6.1; R Foundation for Statistical Computing, Vienna, Austria). Graphs were created with GraphPad Software (GraphPad Prism 8.2.1, San Diego, California, USA). Bonferroni correction was used to account for multiple testing. Reported *p *values are two-sided and were considered statistically significant at *p* < 0.05.

## Results

### Technical validation

**Fig. 2:**
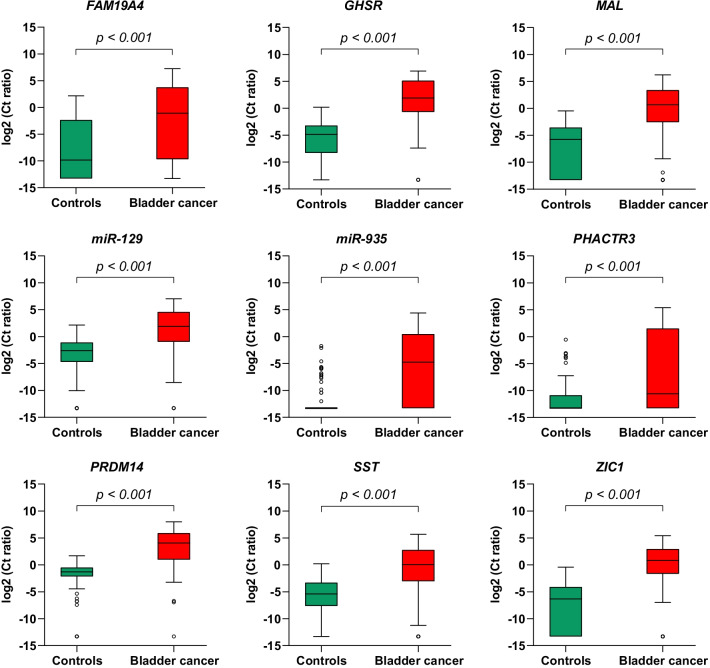
Boxplots of the urinary methylation markers *FAM19A4, GHSR, MAL, miR-129, miR-935, PHACTR3, PRDM14, SST* and *ZIC1*. The *Y*-axis displays log2-transformed Ct ratios of the methylation markers, the *X*-axis divides controls and patients with bladder cancer. Boxes represent medians with 25th and 75th percentiles. Whiskers and outliers are plotted with the Tukey method. *p *values were calculated with the Mann–Whitney *U* test and Bonferroni correction (original *p* value × 9)

**Fig. 3:**
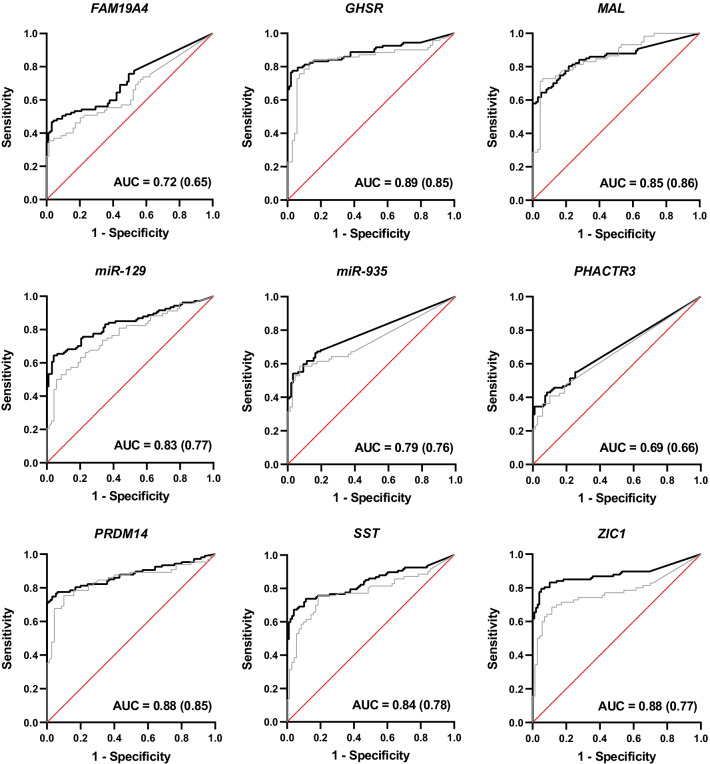
Receiver operating characteristic (ROC) curves of the urinary methylation markers *FAM19A4, GHSR, MAL, miR-129, miR-935, PHACTR3, PRDM14, SST* and *ZIC1*. For each marker, the ROC curves for urine pellet in the present study (black line) and for full void urine in the previous exploratory study (grey line) are visualized. The sensitivity is shown at the *Y*-axis, for 1-specificity at the *X*-axis. The area under the curve (AUC) of the present study is provided in the lower right corner, with the AUC of the previous exploratory study between parentheses [9]

The urinary methylation levels of *FAM19A4, GHSR, MAL, miR-129, miR-935, PHACTR3, PRDM14, SST* and *ZIC1* were significantly higher in patients with bladder cancer than in controls (all, *p* < 0.001) (Fig. 2). To confirm the technical performance of DNA methylation analysis in urine pellet, we compared the AUCs of these nine methylation markers in urine pellet in this independent study population (*n* = 208 participants) to the AUCs of our previous exploratory cohort in which full void urine was used (*n* = 147 participants) [9]. Figure 3 shows a high similarity between the ROC curves of both studies [9]. All methylation markers, except *MAL*, reached a slightly higher AUC in the present study. The AUC of *ZIC1* increased most, from 0.77 in the exploratory study to 0.88 in the present study.

### Preclinical validation

Next, the diagnostic performance of the previously determined optimal marker panel *GHSR/MAL* was assessed [9]. The marker panel reached an AUC of 0.89 (95% CI 0.84–0.94), corresponding to a sensitivity of 80% (95% CI 71–87) and a specificity of 93% (95% CI 85–97) (Table 2). Upon LOOCV, an identical diagnostic accuracy (AUC 0.89) was obtained. A random forest analysis on all nine markers led to a comparable diagnostic performance with a sensitivity of 81% (95% CI 74–89) and a specificity of 93% (95% CI 88–98). This underlines the appropriate selection of *GHSR/MAL* as the optimal marker panel to distinguish between bladder cancer patients and controls.

**Table 2:** Preclinical validation and leave-one-out cross-validation (LOOCV) of the marker panel *GHSR/MAL*

Study population	*GHSR/MAL* Preclinical validation	*GHSR/MAL* LOOCV
*All patients*		
AUC	0.89 (95% CI 0.84–0.94)	0.89
Sensitivity	0.80 (95% CI 0.71–0.87)	0.79
Specificity	0.93 (95% CI 0.85–0.97)	0.76
*Males*		
AUC	0.95 (95% CI 0.92–0.99)	0.95
Sensitivity	0.92 (95% CI 0.83–0.97)	0.90
Specificity	0.93 (95% CI 0.83–0.98)	0.89
*Females*		
AUC	0.81 (95% CI 0.70–0.92)	0.81
Sensitivity	0.79 (95% CI 0.60–0.91)	0.76
Specificity	0.72 (95% CI 0.55–0.85)	0.69

*AUC* area under the curve, *CI* confidence interval, *LOOCV* leave-one-out cross-validation

Results are given for all patients, for males and for females. Based on previous and current differences in the sensitivity of the marker panel *GHSR/MAL* for gender, post hoc analyses were performed with males and females considered as separate cohorts. Hereto, new thresholds of the Ct ratios were determined with Youden’s J index for males and females separately (Additional file 1: Table S1) [16, 17].

The sensitivity of the marker panel *GHSR/MAL* was determined in different subgroups of patients with bladder cancer. These subgroup analyses showed that sensitivity increased with higher tumour grades (G3 vs. G1-G2, HG vs. LG), higher tumour stages (≥ T2 vs. Ta/T1/Tis), in primary vs. recurrent tumours, and in males vs. females (Table 3). Sensitivity of the marker panel *GHSR/MAL* was 59% in females and 89% in males (*p* = 0.001).

**Table 3:** Sensitivity of the marker panel *GHSR/MAL* in different subgroups of patients with bladder cancer

Subgroups	*GHSR/MAL*	*p *value
All (*n* = 108)	80 (71–87)	
*WHO 1973*		< 0.001
G1-G2 (*n* = 56)	66 (52–78)	
G3 (*n* = 52)	96 (85–99)	
*WHO 2004/2016*		< 0.001
LG (*n* = 45)	58 (42–72)	
HG (*n* = 63)	97 (88–99)	
*Tumour stage*		0.007
Ta/T1/Tis (*n* = 85)	75 (64–84)	
≥ T2 (*n* = 23)	100 (82–100)	
*Primary/recurrence*		< 0.001
Primary (*n* = 76)	89 (80–95)	
Recurrence (*n* = 32)	59 (41–76)	
*Gender*		0.001
Male (*n* = 79)	89 (79–94)	
Female (*n* = 29)	59 (39–76)	

*HG* high-grade, *G* grade, *LG* low-grade

Differences in sensitivity between the dichotomized subgroups were analysed with the Chi-square test.

### Post hoc analyses for gender

As the sensitivity of the marker panel *GHSR/MAL* differed substantially between males and females, its diagnostic performance was subsequently assessed with new thresholds for both genders separately (Additional file 1: Table S1). In males, the marker panel reached an AUC of 0.95 (95% CI 0.92–0.99), corresponding to a sensitivity of 92% (95% CI 83–97) and a specificity of 93% (95% CI 83–98) (Table 2). For females, the marker panel yielded an AUC of 0.81 (95% CI 0.70–0.92), at a sensitivity of 79% (95% CI 60–91) and a specificity of 72% (95% CI 55–85). LOOCV reached a similar diagnostic accuracy in both genders (Table 2).

## Discussion

Technical validation of DNA methylation analysis showed that its application to either urine pellet or full void urine is suitable for urinary bladder cancer detection. Highly reproducible results were found between both urine fractions in independent study populations. The robustness of DNA methylation analysis is further supported by preclinical validation of the marker panel *GHSR/MAL.* A cross-validated AUC of 0.89 was obtained, which is consistent with the previous exploratory study [9]. A random forest analysis confirmed that *GHSR/MAL* was rightfully selected as the optimal marker panel to differentiate cases from controls. The marker panel *GHSR/MAL* did not miss any of the muscle-invasive tumours and only two of the non-invasive HG/G3 tumours. Present findings underline the potential of this methylation test for future bladder cancer diagnostics.

In general, for clinical adaption and prevention of unnecessary cystoscopies, urinary biomarker tests should be highly sensitive and specific. For the detection of primary disease, a high sensitivity of urinary biomarker tests is of utmost importance, as the consequences of not detecting a clinically significant bladder cancer may be high. When used in follow-up for recurrence, it might be assumed that a lower sensitivity is acceptable when urinary biomarker tests are alternated with cystoscopies, since an undetected bladder cancer in urine is then noticed on the next cystoscopic evaluation. Of course, the delayed detection of a bladder tumour is less critical in case of low-risk disease compared to high-risk disease. An alternating approach of cystoscopies and urinary biomarker tests could reduce the number of hospital visits and invasive cystoscopies. It is expected that besides improving quality of life of patients under follow-up for recurrence, this approach will also reduce the economic burden associated with bladder cancer.

Our and other urinary biomarker tests performed better in higher tumour grades and stages [18–23]. Four large-scale publications concerning the Bladder EpiCheck test, encompassing 15 undisclosed methylation markers for the surveillance of NMIBC patients, showed that overall sensitivity and specificity ranged 62–68% and 82–88%, respectively [20–23]. However, the sensitivity was higher in HG tumours (79–89%) compared to LG tumours (40–54%). In addition, one of these studies reported that the sensitivity decreased from 100% in T1 tumours to 52% in Ta tumours [23]. The sensitivity of urinary biomarker tests is presumably lower in patients with favourable tumour characteristics (small, LG or low-stage tumours) as these tumours harbour less molecular abnormalities [24, 25]. Diagnostic performance of the marker panel *GHSR/MAL* was also higher in primary than in recurrent tumours. It is expected that bladder tumours have a higher tumour load at primary diagnosis than during surveillance, resulting in a higher sensitivity of the marker panel *GHSR/MAL* in primary tumours. A combined assay of complementary biomarker types might encompass the variability in tumour characteristics [6]. Recently, a prospective study in 838 haematuria patients used a six-marker model consisting of three methylation markers (*ONECUT2, OTX1* and *TWIST1*) and three mutation markers (*FGFR3, HRAS* and *TERT*), as well as the presence of macroscopic haematuria. The combined assay reached 96% sensitivity at 73% specificity for the detection of primary bladder cancer, with 91% sensitivity in LG and 99% sensitivity in HG tumours [26]. Moreover, technological developments that enable the sensitive detection of low amounts of tumour-related material may further increase the diagnostic performance of urinary biomarker tests in all bladder tumours.

Similar to previous studies we found a marked difference between the sensitivity of the marker panel *GHSR/MAL* in males and females, whereas its specificity was similar in both genders [9, 18, 27]. The marker panel performed excellent in males, while its diagnostic performance was good in females. We assume that methylated DNA in urine of females is diluted due to the abundance of unmethylated DNA from normal cells of gynaecological origin [18, 27]. Furthermore, the presence of leucocytes might also cause high background values of unmethylated DNA in urine of females [27, 28]. A possible method to enhance the sensitivity of the marker panel *GHSR/MAL* in females comprises the use of midstream urine, as gynaecological cells are predominantly present in the first void [29]. This approach might prove to be particularly beneficial in female patients with favourable tumour characteristics, in whom the amount of tumour-related DNA in urine is expected to be low [30]. Another possible method to overcome gender-related differences is the use of different thresholds for males and females (Table 2 and Additional file 1: Table S1). The present study was, however, limited by the low number of female participants, hampering any definitive conclusions on this topic.

Other limitations of this study concern its case–control design, which hampers an evaluation of the diagnostic value of the marker panel *GHSR/MAL* in routine clinical practice. Secondly, patients with primary as well as recurrent bladder cancer were included. Thirdly, controls were matched based on age and gender, but not all visited the urology clinic because of bladder cancer symptoms. Although methylation levels did not differ between the control cohorts, specificity might still be overestimated.

Taken together, testing for the methylation marker panel *GHSR/MAL* in urine may provide a valuable non-invasive strategy to detect bladder cancer. Present findings warrant further studies on the clinical value of this methylation test for the primary detection of bladder cancer and in the follow-up of NMIBC patients after TURBT, as a means to reduce the number of invasive cystoscopies.

## Conclusions

Present data support the technical robustness of DNA methylation in urine by demonstrating the suitability of both urine pellet and full void urine for the non-invasive detection of bladder cancer. Preclinical validation of the marker panel *GHSR/MAL* yielded a cross-validated AUC of 0.89, which is identical to a previous exploratory study.

## Supplementary Information

**Additional file 1: Table S1**. Thresholds of the Ct ratios for the marker panel GHSR/MAL in urine pellet, determined by Youden’s J index.

## Data Availability

Data are available upon reasonable request.
