# Supplementary material for: Bladder cancer detection in urine using DNA methylation markers: a technical and prospective preclinical validation
Source: Clin Epigenetics. 2022 Feb 5;14:19. doi: 10.1186/s13148-022-01240-8 (PMC8818199; doi:10.1186/s13148-022-01240-8)
Supplement: Supplementary file 1 — Additional file 1: Table S1. Thresholds of the Ct ratios for the marker panel GHSR/MAL in urine pellet, determined by Youden’s J index. [file 13148_2022_1240_MOESM1_ESM.docx]

| **Marker** | **Thresholds**  **Overall**  (n=108 bladder cancer, n=100 controls) | **Thresholds**  **Males**  (n=79 bladder cancer, n=63 controls) | **Thresholds**  **Females**  (n=29 bladder cancer, n=37 controls) |
| --- | --- | --- | --- |
| *GHSR* | -0,96600 | -1,74985 | -1,02040 |
| *MAL* | -1,45685 | -0,41050 | -4,42380 |

**Supplementary Table 1.** Thresholds of the Ct ratios for the marker panel *GHSR/MAL* in urine pellet, determined by Youden’s J index.^1, 2^ Based on previous and current differences in the sensitivity of the marker panel *GHSR/MAL* for gender, post-hoc analyses were performed with males and females considered as separate cohorts. Hereto, new thresholds of the Ct ratios were determined with Youden’s J index for males and females separately.

**REFERENCES**

1. Youden WJ. Index for rating diagnostic tests. Cancer. 1950;3(1):32-5.

2. Schisterman EF, Perkins NJ, Liu A, Bondell H. Optimal cut-point and its corresponding Youden Index to discriminate individuals using pooled blood samples. Epidemiology. 2005;16(1):73-81.
